# Adjunctive Testing Using Biospectral Emission Sequencing: Bioregulatory Intelligence Technology in Parallel With the Goals of Artificial Intelligence in Medicine

**DOI:** 10.7759/cureus.65739

**Published:** 2024-07-30

**Authors:** David A Jernigan

**Affiliations:** 1 Complementary Medicine, Biologix Center for Optimum Health, Franklin, USA

**Keywords:** electromagnetic signatures, raman spectroscopy, machine learning, bioregulatory intelligence, artificial intelligence, biospectral emission sequencing

## Abstract

The many advancements in medical technology of the last century have continually sought to improve the sensitivity of testing and the specificity of treatment of human maladies. Conventional physical and pharmaceutical treatment is largely an imprecise process, stimulating the impetus for the advancement of machine learning-enhanced artificial intelligence (AI) medical technologies. Biospectral Emission Sequencing (BES) is a bioregulatory intelligence (BI) technology already in use as an adjunct to conventional testing. Biospectral Emission Sequencing provides a functional system of dynamic real-time adjunctive testing and treatment selection. This paper discusses the parallel technologies of present and future AI and BI technologies in medicine.

## Introduction and background

Biosensing in medicine: artificial intelligence (AI) and bioregulatory intelligence (BI)

The greatest challenge in the healing arts is to develop better ways to diagnose and select the best treatments. The gold standards of blood and radiological testing have the limitations of being slow and often imprecise in their diagnostic conclusions. Treatment selection using this model of testing is generalized to symptom-relieving therapeutics since the causes of the illness are infrequently identified. Advancements in AI and BI technologies are showing great promise toward achieving real-time functional precision diagnostics and treatment selection. Both AI and BI technologies share many of the same goals yet operate from parallel paradigms.

Artificial intelligence in medicine is various technologies often combining computer-based testing and machine learning with nano-enabled biosensing. These advancements in AI enable smart drug delivery technologies and the manipulation of matter, as in tissue engineering, via self-organizing and self-assembling nanoparticles, nanopolymers, and hydrogels [1-3].

In contrast, BI in healthcare is a functional, non-computer-based biosensing technology of the coherent, biogenic, optogenetic bioinformation system of the human body [4-7]. Various technologies have been developed that have advanced BI testing; possibly the most advanced is Biospectral Emission Sequence Testing (BES), a technology that enables the practitioner to access the electromagnetic signatures (EMS) emitted by DNA and the human organizing principles for the purpose of precision, real-time, adjunctive diagnostics and treatment selections that work in harmony with the natural structure and function of the body.

The goal of both medical AI and medical BI technologies is to enable rapid and dynamically flexible testing by accessing the biologically coherent information stored and emitted by DNA and the human body. Using BI technologies, it is becoming possible to rapidly identify structural and functional issues and interferences to normal bioregulation within the human body. Bioregulatory intelligence technologies, such as BES, are able to go beyond the detection of issues and can, through the sequencing of EMS, determine biogenically guided, precision treatments that address pathology at the causal levels. Rapid symptom relief and resolution often follow as the result of facilitating the elimination of interferences and restoring the optimum structural and functional integrity of the human organism. The utility of these new BI advancements is that they eliminate much of the guesswork that is common in the clinical setting regarding diagnosis and treatments. The new BI-enhanced precision adjunctive diagnostics are real-time testing that can identify and prioritize the importance of anything interfering with the optimum structural and functional integrity of the body. Bioregulatory intelligence-enhanced treatment selection can determine the optimum natural or prescription medications that will work in synergy with the body and can determine the optimum tolerated dosage, the duration of treatment, as well as the potential of unintended secondary effects, such as the potential of the patient being allergic to the treatment or the treatment having a toxic effect on the body, prior to administering the treatment. 

Bioregulatory intelligence technologies represent a clear shift away from the management of symptoms with lifetime medications and canned treatment protocols to a more causal focus with clear guidance to their associated issues. Precision, seamless real-time testing through treatment selection brings healthcare closer to the goals of rapid and long-term unmedicated symptom relief and the true restoration of quality of life. 

Bioregulatory intelligence technologies applied to healthcare research are a contender in discovering heretofore unknown causes of illnesses, which, when combined with precision treatment selection, can lead to the body’s ability to restore optimum health even in previously treatment-resistant illnesses.

The present state of the science of AI and BI technologies

Albert Einstein is widely credited as having said, “Future medicine will be the medicine of frequencies” [8]. Everything in the universe is a vibration within the electromagnetic spectrum. The renowned inventor Nikola Tesla is quoted as saying, “If you want to find the secrets of the universe, think in terms of energy, frequency, and vibration.” These quasi-prophetic statements are coming to pass with the advent of AI and BI technologies. In more contemporary perspectives, Douglas C. Wallace comprehensively helps us understand how internal and external sources of energy, frequency, and vibration can indeed influence dynamic adaptation in the case of maintaining health or maladaptation in cases of illness. Wallace states, "Medicine pertains exclusively to humans, a single species. Consequently, the most important variables in intraspecific adaptation to local environmental changes, which are primarily energetic, should be alterations in bioenergetic genes, either genetic or epigenetic. Mutations in mtDNA are more common than nDNA mutations, and epigenomic changes can rapidly change the expression of bioenergetic genes. Efforts to explain common “complex” diseases like diabetes based exclusively on the analysis of nDNA variation in tissue-specific genes would then be expected to be relatively unproductive, as has been the case. In reality, common diseases may not be particularly “complex”; they may simply be energetic and non-Mendelian [9].

The leading edge of computer science research is turning to the electromagnetic information storage capacity of human DNA for inspiration. The U.S. government has launched an initiative called the Molecular Information Storage Program, where it is reported that the storage of information will soon be in the form of synthetic DNA. In their article titled "DNA: The Ultimate Data-Storage System", Ionkov and Settlemyer state, “DNA can archive a staggering amount of information in an almost inconceivably small volume. Consider this: humanity will generate an estimated 33 zettabytes of data by 2025, that is, 3.3 followed by 22 zeroes; DNA storage can squeeze all that information into a ping-pong ball, with room to spare. The 74 million-million bytes of information in the Library of Congress could be crammed into a DNA archive the size of a poppy seed-6,000 times over. Split the seed in half, and you could store all of Facebook’s data” [10].

Synthetic DNA information storage is apparently well on its way to functionality. The Los Alamos National Laboratory has developed a way to transfer the usual binary code of computers to the structure of synthetically made DNA by encoding variations of the four base nucleotides that make up DNA: adenine, guanine, cytosine, and thymine. Variations of these four nucleotides make up the genetic codes or blueprints that enable the instructions for building every living thing on the planet. This advancement will transform the computer world and especially the development of biomedical artificial intelligence technologies. Again, this is being done completely synthetically, following the same form as human DNA. This technology reveals the amazing computing power of human DNA and its ability to store and transmit information. Bioregulatory intelligence technologies, such as BES, seek to access this stored and emitted bioinformation within the human body.

Many are shocked to learn that every strand of human DNA is a bit over six feet long. If one were to stretch out, end to end, all the trillions of DNA in one human body, it would be 65 billion miles long [11]. That is enough DNA in one human body to take 150,000 round trips from Earth to the moon. When considering the profound amount of data storage capacity in a small amount of synthetic DNA, the even greater information storage capacity of the DNA in a person’s body is quite staggering. It is known that human DNA functions via biophotonic and other electromagnetic emissions; DNA is a transceiver, transformer, and transmitter through which bioinformation flows [12]. It is this bioinformation that is accessed by the BI technology, BES.

Medical AI technologies and BI medical technologies are parallel systems developed to access the previously inaccessible bioinformation stored in each person’s body. These technologies are poised to drive diagnostic and treatment determination in healthcare.

The present state of AI is advancing with reports of the potential of enabling the more precise matching of cancer drugs to patients. In a study of 41 patients with malignant melanoma and 33 patients with breast cancer, if the patient had just one problematic clone, then the patient would not respond to the drug [13]. 

**Figure 1:**
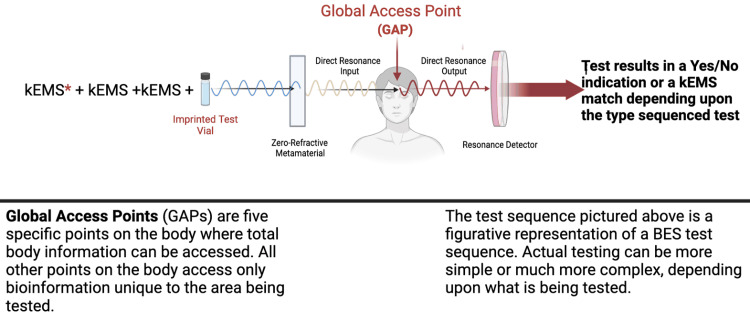
The use of multiple individual known electromagnetic signatures in combination with an imprinted specimen vial in biospectral testing of Biospectral Emission Sequence (BES) testing for determining more specific information concerning a patient Although this depiction seems linear, the reality is both the input and the resulting resonance frequency intercept above the metamaterial, which is placed over the global access point located on the patient between and above the brow. In this example, known electromagnetic signatures are used in combination with an imprinted test vial, much like words in a sentence ending in a question mark, in a Biospectral Emission Sequence test to determine more specific information throughout the entire body. These known electromagnetic signatures (EMS) are either biogenic digitally generated signatures or generated via a modulated microcurrent device to result in a binary yes/no response as determined by the presence or absence of a harmonic resonance when using the Kapton resonance device. The zero-refractive index metamaterial reduces noise and enhances the detection of the EMS being targeted. In this manner, bioinformation can be determined using a range of sequenced assays in the form of electromagnetic sentences to determine an almost limitless range of issues with the patient's body. This sequencing of signatures can be structured in complex flowcharts to find the location or origin from which a symptom in the patient is arising either locally or remotely to where the symptom is felt, what the exact issue is, and then the identification of what the patient's unique body would need to correct the issues that were identified. The author created the figure using BioRender (BioRender, Toronto, Canada)

The present state of BI testing is highly dynamic, enabling real-time testing of a patient in either direct resonance testing as presented in Figure 1, enabling adjunctive diagnosis of specific infections or pathologies, or BES testing can be utilized in sequenced testing in variable yes/no flowcharts to go from where a pathology or symptom is arising to what is the primary causative issue, to the ultimate treatment for that unique issue and patient. Biospectral Emission Sequencing flowcharting in this manner can go beyond the primary level of involvement to identify any deeper layers of associated causative agents in local or remote locations in the body.

**Figure 2:**
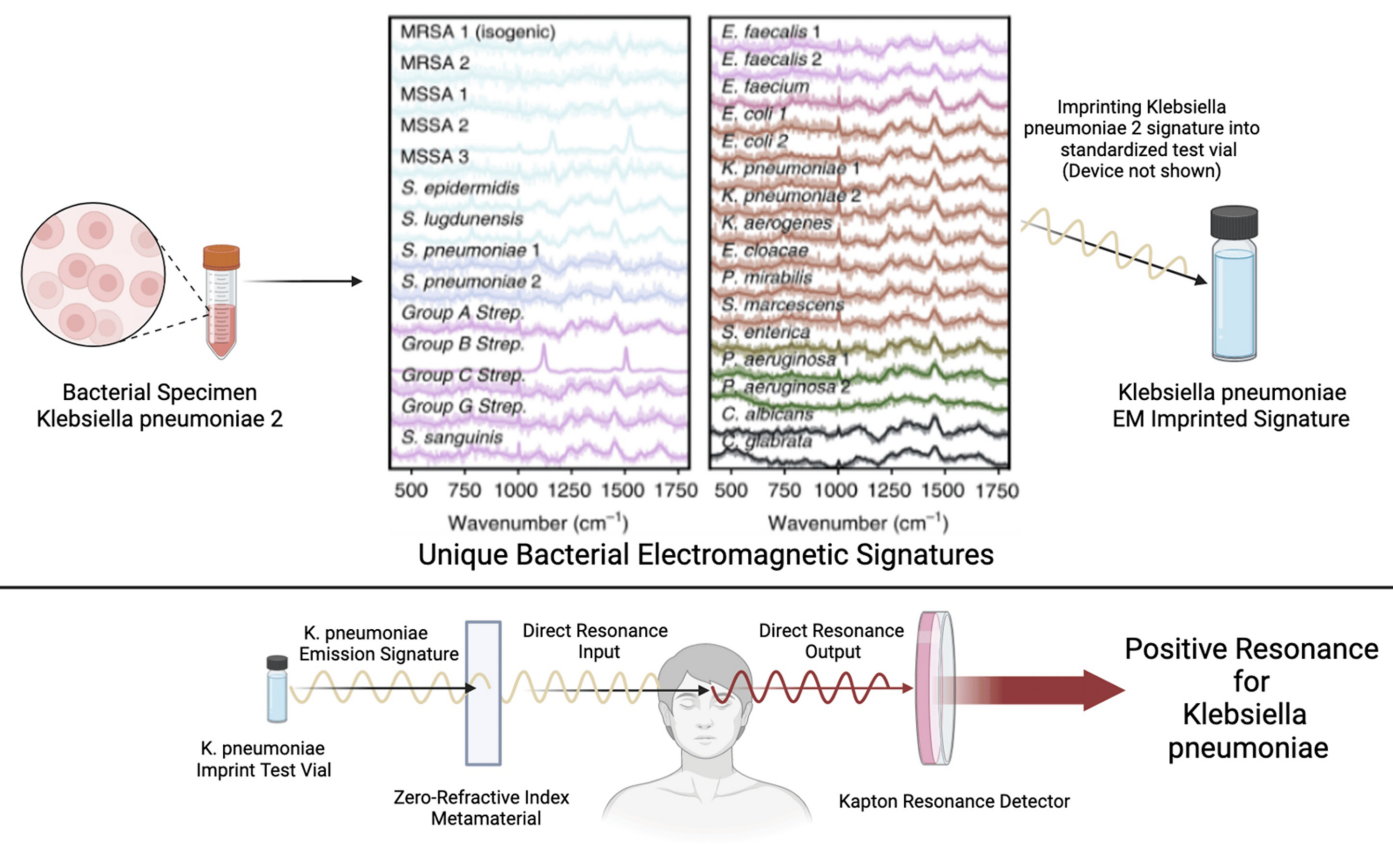
The imprinting of the electromagnetic signature of a biological specimen for use in Biospectral Emission Sequence (BES) test for infection in a patient This figure depicts how a test vial is created for the purpose of being used in the sequencing of the challenge sequence in BES testing. Starting with a bacterial specimen, in this example, *Klebsiella pneumoniae* (*K. pneumoniae*), an imprint of the bacteria’s emitted electromagnetic signature (EMS) is created by a digital electromagnetic imprinting device (not pictured) into a test vial. The test vial contains a graphite rod, which permanently stores the imprint. The upper central graph represents a pictorial representation of the known EMS of various species of microbes. The emitted EMS from the vial of the *K. pneumoniae* specimen is used to imprint the test vial. The graphic representations of bacterial strains are a visual representation of the otherwise imperceptible EMS emitted by various types of bacteria, as detected by classic frequency Raman spectroscopic analysis. b. An example of using a *K. pneumoniae*-imprinted test vial (the imprinted vial contains only the EMS of the bacteria) in a sequenced BES test, specifically called a direct resonance test. The *K. pneumoniae*-imprinted vial emissions are used over a zero-refractive index metamaterial to minimize the noise and enhance the coherence of bioinformation being emitted from the patient's body at the Global Access Point site located slightly above and between the eyes on the brow. The test is considered positive if a harmonic resonance is detected by the Kapton resonator device between the emitted electromagnetic signature of the imprinted *K. pneumoniae* specimen vial and that of the identical emissions radiating out of the patient's body from the *K. pneumoniae* infection. The author created the figure using BioRender (BioRender, Toronto, Canada)

Perhaps the most representative example of the potential for BI as used presently in healthcare is the compelling research that was published using BES in the adjunctive testing of 26 individuals. In this case, BES was used as an adjunctive diagnostic tool and also to develop a treatment solution. In this study, BES testing correctly identified the presence of one or more *Borrelia* sp. infections in a similar manner as detailed in Figure 1 and Figure 2. The BES findings were subsequently verified by the recently developed and highly accurate laboratory test, the Phelix Borrelia-Phage test. This test is reported as having a 96% sensitivity and 100% specificity in acute, chronic, or antibiotic-resistant infections [14].

Biospectral Emission Sequencing was also used in the development of treatment solutions in each of the 26 individuals by sequencing specific energetic signatures with the goal of inducing naturally occurring, native, polyvalent bacteriophages to eliminate the specific strain of *Borrelia *identified in each of the 26 individuals. Post-treatment testing with the Phelix Borrelia-Phage test resulted in the apparent elimination of the targeted infection in 92% (24 of the 26) of the individuals. The initial BES adjunctive diagnostic testing and subsequent apparent complete elimination of the targeted infection, combined with clinical improvements in the patients, appear to support the working hypothesis. As discussed above, the BES-sequenced energetic imprints enabled the induction or alterations in bioenergetic genes, either genetic or epigenetic, in the types of native polyvalent bacteriophages that would and could be induced to turn virulent and kill the targeted *Borrelia *strains. It is important to note that in cases where the individuals tested positive for two different strains of *Borrelia*, such as *Borrelia burgdorferi* and *Borrelia miyamotoi*, only one strain at a time was targeted. Upon retesting using the Phelix Borrelia-Phage test, only the targeted strain was tested as eliminated, while the untreated strain persisted. When this latter strain was targeted using the BES to strategically induce other polyvalent phages to eliminate the remaining strain of *Borrelia*, repeat Phelix Borrelia-Phage testing resulted in it being apparently eliminated [14]. No adverse effects of the treatment were reported, nor was there a typical Jarisch-Herxheimer reaction worsening of the patient's symptoms due to the speed of bacteriophage killing of the targeted infection and the absence of bacterial toxins in Borrelia spirochetes [15]. To date, outstanding clinical results have been seen in many types of bacterial, viral, fungal, mycoplasmal, and other types of infections.

The individual energetic imprints targeting the unique strains of *Borrelia *in the study mentioned above were studied to detect each formulation's unique energetic signature when compared to each formulation and a control. Each imprinted formulation was found to be unique from the next and stable since the formulations were each produced three years prior [16].

Although historically healthcare is a field that is highly resistant to such radical changes, BI and AI in present-day use demonstrate profound improvements are making their way into the delivery of precision diagnostics and individualized treatment selection in some of the more advanced healthcare facilities.

Healthcare in the near future

The mainstream doctor of the future is predicted to be guided by machine learning-enhanced AI to address issues within the human body before they manifest into serious diseases. Machine learning is the part of AI that enables it to catalog, store, and process meaningful data gathered from the test subject in the form of EMS without the need to stay within its explicit programming.

Researchers Qi and Hu, et. al., published impressive compilations of research where machine learning-assisted AI has demonstrated its diagnostic ability, diagnosing with very high sensitivity and specificity in a wide range of infections, cancers, and other illnesses [17]. Artificial intelligence is predicted to enable direct access, not only to access a person’s complete medical records but also to access their real-time bioinformation, through interfacing with nano-enabled biosensing systems and the Internet of Medical Things (IoMT) [18,19]. Microbiologist Alex Berezow, PhD, reported in his article on the educational website for the American Council on Science and Health, citing several peer-reviewed article references suggesting the likelihood of artificial intelligence soon replacing pathologists, radiologists, and microbiologists due to AI’s greater accuracy, sensitivity, and speed [20].

It is indeed likely that human-performed laboratory testing of the blood and many special imaging tests will become obsolete, as will the need for the physician to interpret the findings when those tests are utilized. A visit to a healthcare professional will be guided either by AI and/or BI technologies, which will scan the patient’s body, detecting and gathering biological data as well as determining the functional diagnosis and the most ideal treatments [21,22]. Although recent articles attempt to assure readers that AI will complement, not replace, physicians, this sentiment is likely designed to allay fear in the medical community of being replaced [23].

Bioregulatory intelligence technologies are already in clinical use, making significant leaps forward in the last 30 years to its most advanced testing, BES.

This author predicts that future advances in BI technology will enable the in vivo bioproduction of synthetic medication or biologically identical substances by inducing the human or native microbial genomic engine via complex EMS sequencing. Other advancements will likely be in the field of regenerative medicine, whereby, through EMS sequencing, blastic cells within the extracellular matrix can be induced to revert into pluripotent stem cells, which can be further directed to repair and regenerate the strategically targeted tissue.

## Review

The application of BES testing

Biospectral Emission Sequence technology is a biogenic and biophotonic system of testing and treatment based upon the general principles of molecular resonance frequency-Raman spectroscopic (FRS) techniques. Named after physicist C. V. Raman, frequency-Raman spectroscopy utilizes photonic resonance interactions with the molecules in materials, causing an inelastic scattering of light emissions and the unique electromagnetic signature of the molecular structure of the substance being tested. The FRS technique is used in applications of chemical and biological sensing and microbial identification within complex materials.

Similarly, as in FRS, BES enables the sequencing of these EMS into coherent sentence structures, enabling true real-time bioinformation integration between the practitioner/patient. Biospectral Emission Sequence makes it possible to detect positive resonance in a biogenically sequenced signal challenge of a sample or person. In BES testing, to determine the presence of a particular pathogen, a sample of the pathogen or its kEMS imprint can be used in a test sequence into one of five Global Access points (GAP), and using the Kapton device, the practitioner can read changes in resonance, indicating the presence or lack of the EMS for the pathogen as a resonance emission from within the patient’s body (Figure 2). The GAPs are five documented points on the human body that enable access to the bioinformation of the total body when performing BES. The sensing aspect of a positive BES resonance test is performed using a polymeric Kapton resonance membrane. Kapton is widely used in industrial/military sensing devices for chemical and biological sensing applications [24].

The transmission of sequenced challenge frequencies in BES testing results is binary, either resulting in a negative or positive resonance via the Kapton resonance detection device. This positive or negative test is viewed as a yes or no for the tested issue. Unlike FRS, which transmits a single determined light frequency to challenge a molecular resonance interaction of a target within a substance, BES transmits complex sequences of EMS into the body to interact with targeted molecules and bioinformation within the patient’s body. The positive BES resonance between the test sequence and the targeted molecular frequencies of cells, microbes, pathologies, or tissues adjunctively indicates the likely presence of the known transmitted challenge signature from within the test subject (Figure 2).

Similar technologies to BES are being used in laboratory settings, such as conventional UV-resonance Raman sequence analysis, which is used for the detection and differentiation of microbial types and strains in medical microbial testing, and even in mixed background materials such as soil and food [25]. For example, Raman spectroscopic photonic analysis is used for the rapid identification of microorganisms based on their unique coherent molecular and DNA emissions [26]. These emissions are enhanced by the liquid-crystalline matrix found in living systems. It is believed BES also induces conventional biogenic-sequenced, resonance Raman-like challenges through global (GAP) bioinformation points on the body to characterize specifically tested microbial cells, as well as resonance Raman excitation in the UV-range to obtain selectively enhanced signals of the microbial DNA/RNA and aromatic amino acids when the targeted microbial strain or targeted pathology is present within the body [27]. The positive resonance between the challenge frequency of a known microbial strain and the same strain within the body is historically associated with a high sensitivity and high specificity [28,29]

The material being tested emits the full-spectrum EMS comprising its molecular and DNA structure, enabling multiplex targeting of biological actions against various sequenced targets.

In this regard, BES functions as a real-time assay of the electromagnetic fields that contain the myriad of frequencies emanating from the tissues and molecules in the body. Xiuxiu Wang et al. report, “Some surveys found that changes of external electromagnetic field could cause different biologic effects. For a certain frequency, a biologic electromagnetic resonance phenomenon could occur in a specific biologic site. For instance, the visible region of the electromagnetic field can cause energy level transitions of biologic macromolecules; the infrared and millimeter-wave region of the electromagnetic field can cause vibration and disturbance of biologic macromolecules, facilitating structure conformational change; and the extremely low-frequency region of the electromagnetic field leads to the polarization of biomedium, changing the charge distribution and conduction. These results confirmed the existence of an intrinsic electromagnetic field and the different physiologic and biochemical reactions corresponding to different electromagnetic frequencies” [30].

Similarly, Michael Levin discusses in detail the research documenting the native bioelectric states leading to biochemical signaling and cellular changes that are nonlocal because voltage patterns propagate [31,32]. The difference in discussing bioelectrical changes in physiological conformation and BES is that bioelectric devices, so-called electroceuticals, are concerning on/off electrical stimulation of strategic nerves; however, BES is quite unique in that it is a non-invasive, programmable coherent electromagnetic bioinformation being carried in the emitted signal that transparently interacts with the coherent bioinformation of the targeted issue in the human body.

Biospectral Emission Sequence: a sequenced frequency Raman-like spectroscopy

Electromagnetic signatures can be considered the largely unrecognized language of the universe. Indeed, the universe is likened to a progressive learning hologram, where information is embedded yet also exists independently of the whole [33].

The principle of self-organization and self-assembly that is well recognized today in scientific literature speaks to this concept, the authors of which state, “The science of self-assembly has undergone a radical shift from asking questions about why individual components self-organize into ordered structures to manipulating the resultant order. However, the quest for far-reaching nanomanufacturing requires addressing an even more challenging question: How to form nanoparticle structures with designed architectures without explicitly prescribing particle positions” [34].

The imponderable nature of EMS forming a language or regulating principle in living systems can be challenging to some. The development of BES enables reproducible, real-time testing with high sensitivity and specificity, which is often verified by conventional blood testing or other special testing in healthcare. It utilizes biogenically produced EMS as one utilizes words in language and strings together the EMS to form coherent EMS sentences. When the biogenic EMS is unknown, a test vial of the EMS of an actual substance can be used in the sequencing of the test, as seen in Figure 1.

Electromagnetic signatures can be viewed and used like words are in any language. The EMS signatures are an electromagnetic vocabulary, which can be sequenced into meaningful EMS sentences. The sequencing of EMS enables a wide range of very specific questions that can be posed to the test subject’s bioinformation system. Conventional Raman spectroscopy challenges the EMS of the material being tested, and the resulting resonance results in a very specific, complex, and high-specificity EMS for the type of microbe or substance being tested. Once the resonance EMS for the identified microbe is known, it is added to the standardization database for future diagnostic use [35].

As the BES vocabulary increases and the skill of posing better questions in the form of sequenced sentences improves, the diagnostic and treatment specificity improves. The adage "ask a better question, get a better answer" applies here. Because BES is a real-time test performed dynamically by the healthcare provider, the experience and knowledge can limit the application of the test. The possibilities of testing are almost limitless, and because of this reality, very good sequence testing can be performed perfectly; however, it still might not get to the most problematic issues that need to be addressed. To compensate for these possible limitations, general testing protocols are established to ensure a more ideal set of sequences, much like sequential flowcharting is performed to ensure the priority issues causing the patient’s illness are determined and the most ideal corrections are initiated.

The biogenic kEMS sequence testing of BES appears to be limitless in its range of capabilities and future possibilities. Findings beyond the scope of this article suggest BES can be used for tissue regulation, regeneration, manipulation of genetic single nucleotide polymorphisms, and other physiologically advantageous targets. Biospectral Emission Sequence accesses the realm of frequencies that make up physical matter and regulate life itself. The information and bioregulation of life in all its forms are controlled via the subtle language of frequencies.

In BES, a resonance emission is set up between the test-sequenced EMS, the tester, the resonance emission, and the Kapton membrane. As in classic Raman spectroscopy, the detection of the laser frequency through the test subject and the resulting stimulated Raman spectroscopic imaging is achieved by spatial frequency multiplexing and single photodiode detection [36]. In BES, the Kapton membrane resonance is analogous to the photodiode detection. Processing of the finding of the positive testing is determined by the parameters of the formatted sequence being tested.

Once the EMS of a type of microbe, such as *Klebsiella pneumoniae*, is identified and cataloged, adding to the kEMS vocabulary, it can be used in a sequenced EM sentence to challenge the patient’s bioinformation system to determine if that specific strain of *Klebsiella pneumoniae* is not just part of the microbiome of the patient’s body but indeed is a priority issue affecting their body (Figure 1).

Limitations

The limitations of AI, according to some, are the lack of transparency in the algorithms used in the diagnostic process [37]. Both AI and BI struggle with physician and patient acceptance of the process and delivery of the technologies. Other inherent limitations of BES testing include that the results are only as good as the known sequences and EMS vocabulary, the arrangement being sequenced, and the accuracy of the EMS signatures being utilized. As in human language, where on a daily basis a person can use a relatively limited vocabulary yet dynamically change the meaning of the sentence being communicated, so can BES test sequencing have the flexibility to use a limited EMS vocabulary of just over 1,100 kEMS signatures to achieve a wide range of test sequences, although the potential is limitless as all substances have unique EM signatures based upon their atomic and molecular structure. Another limitation is common in all testing, one will only get the answer to the question being asked. Many great tests can be performed using BES, and yet if those tests are not asking the right question, resulting in the primary cause of the illness, then it can be challenging to determine what sequence of testing is required to identify the true cause, resulting in symptom resolution. In BES, the test being sequenced can be very broad or extremely specific tests to improve treatment outcomes. It is expected that the vocabulary will exponentially expand as the technology advances.

## Conclusions

Both medical AI technologies and BI technology, BES testing, seek to help eliminate the guesswork common in healthcare to facilitate better and more rapid treatment outcomes. Biospectral Emission Sequencing enables very precise, adjunctive diagnostic testing and precision-tailored treatments at the causative level of the patient’s illness.

Biospectral Emission Sequencing offers a functioning system and technology that enables much-improved acquisition time of diagnostically relevant bioinformation with subsequent improvements in precision treatment identification, tailored to the individual. The real-time testing of BES appears to be superior to conventional systems of blood analysis and treatment selection.

Steps needed to advance these systems of diagnosis and treatment would involve controlled independent studies and continued conventional laboratory confirmation of the accuracy of the diagnostic testing utilizing AI and BI technologies. Beyond the scientific validation, increasing public awareness and acceptance will go a long way to being able to leave behind archaic blood testing systems and treatment selections based on popular usage.
